# Optical imaging spectroscopy coupled with machine learning for detecting heavy metal of plants: A review

**DOI:** 10.3389/fpls.2022.1007991

**Published:** 2022-10-24

**Authors:** Junmeng Li, Jie Ren, Ruiyan Cui, Keqiang Yu, Yanru Zhao

**Affiliations:** ^1^ College of Mechanical and Electronic Engineering, Northwest A &F University, Yangling, China; ^2^ Key Lab Agricultural Internet Things, Ministry of Agriculture & Rural Affairs, Yangling, China; ^3^ Shaanxi Key Laboratory of Agricultural Information Perception and Intelligent Service, Yangling, China

**Keywords:** Heavy Metals, Machine learning, Optical Imaging, Plant, Spectroscopy

## Abstract

Heavy metal elements, which inhibit plant development by destroying cell structure and wilting leaves, are easily absorbed by plants and eventually threaten human health *via* the food chain. Recently, with the increasing precision and refinement of optical instruments, optical imaging spectroscopy has gradually been applied to the detection and reaction of heavy metals in plants due to its *in-situ*, real-time, and simple operation compared with traditional chemical analysis methods. Moreover, the emergence of machine learning helps improve detection accuracy, making optical imaging spectroscopy comparable to conventional chemical analysis methods in some situations. This review (a): summarizes the progress of advanced optical imaging spectroscopy techniques coupled with artificial neural network algorithms for plant heavy metal detection over ten years from 2012-2022; (b) briefly describes and compares the principles and characteristics of spectroscopy and traditional chemical techniques applied to plants heavy metal detection, and the advantages of artificial neural network techniques including machine learning and deep learning techniques in combination with spectroscopy; (c) proposes the solutions such as coupling with other analytical and detection methods, portability, to address the challenges of unsatisfactory sensitivity of optical imaging spectroscopy and expensive instruments.

## Introduction

**Figure 1:**
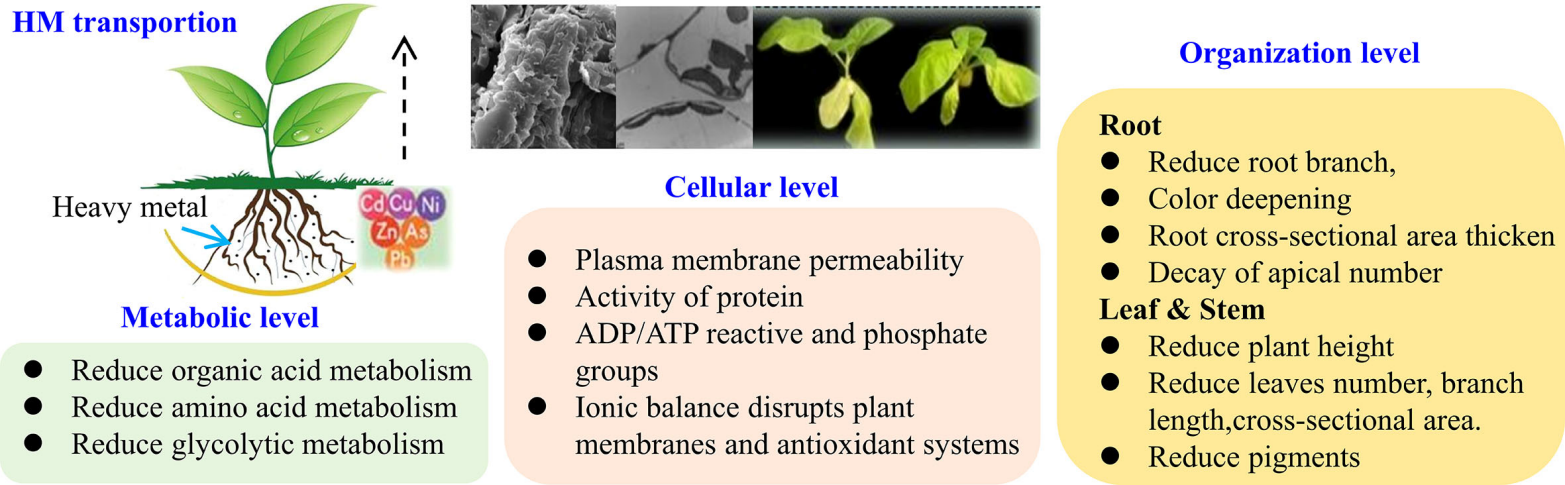
The effects of heavy metals for plant from metabolic, cellular, organization level.

Heavy metal elements are absorbed by plants and induce nuclear and chromosomal aberrations by altering the permeability of the plasma membrane. More precisely, heavy metals can bind proteins and alter their activity, bind to ADP/ATP reactivity and phosphate groups, and interfere with ion homeostasis to disrupt membranes and antioxidant systems, induce nuclear and chromosome aberrations, inhibit cell division, induce cell cycle arrest, and changes cell wall structure. Thus, causing severe damage to plants at the cellular and tissue levels, ultimately leading to stunted plants and reduced crop yield and quality (Liu et al., 2017; Xie et al., 2018; Feng et al., 2021). **Figure 1** illustrates the effects on heavy metals stress (HMS) for the plant from the metabolic, cellular, and organization levels. Moreover, heavy metals threaten human health through the food chain. Therefore, heavy metal contamination is closely related to agricultural production, environmental protection, and food safety (Wang et al., 2018a; AbdElgawad et al., 2020; Yuan et al., 2020).

There are many reliable methods to detect heavy metal contamination in plants, including chemical analysis, electrochemical anodic stripping voltammetry, ultraviolet visible spectrophotometry (UV) (Brzezicha-Cirocka et al., 2016; Wang et al., 2018b), high performance liquid chromatography, atomic absorption spectrometry (AAS) (Ghanati et al., 2019; Li et al., 2021a), inductively coupled plasma mass spectrometry, inductively coupled plasma optical emission spectrometry (ICP-OES). Although these methods have high detection accuracy, they are time-consuming, laborious, complicated sample pretreatment and have a limited detection range, which cannot meet the requirements of green, rapid, and large-scale detection (Bian et al., 2020; Shojaei et al., 2021; Zhao et al., 2021). Recently, with the development of optical instruments and machine learning, the sensitivity of optical imaging spectroscopy has been improved and gradually applied to the detection of heavy metals for plants because of the simple, rapid, and *in situ* advantages provided by spectroscopy (Zhou et al., 2020a; Park et al., 2022).

This review focuses on the application of optical imaging spectroscopy combined with machine learning for heavy metal detection for plants in the last decade or so (2012-2022). The feasibility of using spectroscopy for heavy metal detection is explored from plant roots, stems, leaves, and fruits. In addition, the basic principles of spectroscopy combined with machine learning to detect heavy metal contamination levels in plants are elucidated. Meanwhile, future research orientation and focus are critically proposed to address the difficulties and challenges in the present spectroscopy and artificial neural network development stage.

## Theoretical basis of spectroscopy

Heavy metal elements entering plants damage the cell’s plasma wall structure and affect the metabolism and phenotypic information of the plant (Feng et al., 2021). Therefore, the spectroscopy is divided into molecular and atomic spectra to detect plants according to different responses to heavy metals. Rathod et al. (Rathod et al., 2015) and Jabbar et al. (Jabbar et al., 2019) used atomic and molecular spectroscopy to detect heavy metal stress in plants. Alvarez-Mateos et al. (Alvarez-Mateos et al., 2019) and Aldakheel et al. (Aldakheel et al., 2020) combined traditional techniques and spectroscopic to study heavy metals in plants. It has been a widespread tendency to reveal heavy metal contamination levels of plants from different scales by multi-device/technology linkage. In addition, the combination of spectroscopy and deep learning dramatically improves the efficiency and accuracy of detection, enabling label-free, *in-situ*. Zhang et al. (Zhang et al., 2022) and Zhao et al. (Zhao et al., 2022) et al. used spectroscopy combined with deep learning to study heavy metals in plants.

**Figure 2:**
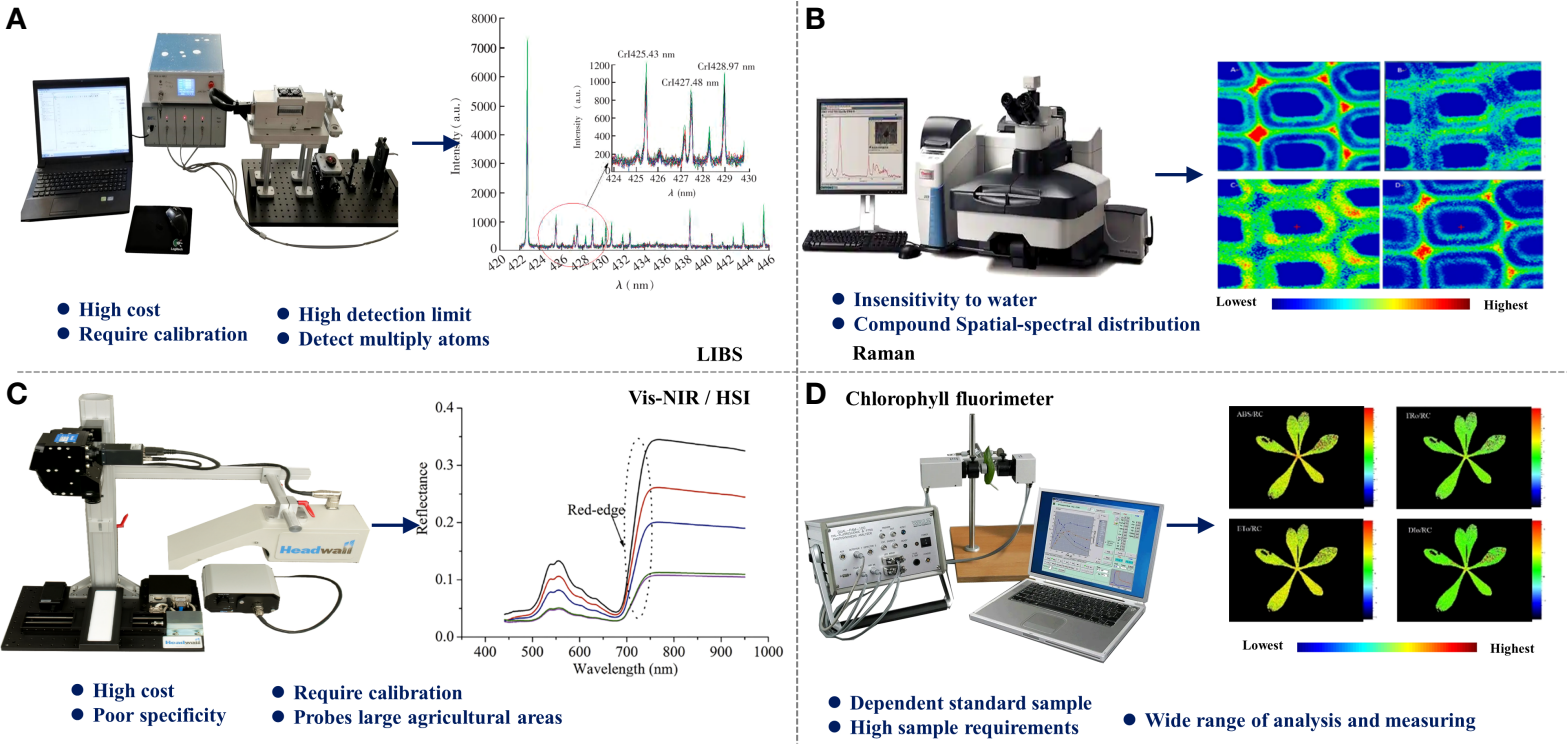
The principle and characteristics of spectral technology. **(A)** Laser induced breakdown spectroscopy (LIBS). **(B)** Raman spectrum. **(C)** Visible/Near-infrared spectroscopy (Vis-NIR)/Hyperspectral imaging (HSI). **(D)** Chlorophyll fluorimeter.

Moreover, heavy metals are absorbed by plant roots and transported to the aboveground organs of plant tissues, which are usually enriched in leaf vacuoles. However, different plants have different absorption and transport abilities for heavy metals, so plants with strong absorption abilities are selected for heavy metal phytoremediation (Feng et al., 2021), thus the combination of optical imaging spectroscopy with temporal and spatial information of plants to construct a multidimensional visual kinetic model to assess the distribution characteristics of heavy metals in plants is a novel approach, which plays a theoretical reference value for breeding and green remediation. **Figure 2** briefly depicts the characteristics of five spectroscopic techniques applied to detect heavy metals in plants.

### Molecular spectrum

Visible and near-infrared spectral data are typically obtained by point scanning in a wide wavelength range (Shen et al., 2020). Vis-NIR spectrometers with the point scanning method are easily carried in the field or on-site detection for their small volume and high reliability (Kawamura et al., 2021). However, due to the influence of overtone and combined vibration, the absorbance characteristics in the visible near-infrared spectrum are usually low (Chen et al., 2021). HSI combined imaging and spectroscopy techniques to obtain high-resolution spectral-spatial information in visible and near-infrared regions (Jun et al., 2019). HSI could provide accurate exploration and visually express plant phenotype information of samples at the pixel level (Shen et al., 2020; Ryckewaert et al., 2021). However, thousands of spectral bands can result in data redundancy, optical complexity, and spatial heterogeneity (Pyo et al., 2020).

Raman spectrum is a kind of molecular scattering based on the inelastic scattering of incident light. The scattering spectra of different incident light frequencies are analyzed to obtain the vibration and rotation information of the molecule and to study the structure of the molecule. Raman spectroscopy has superior spatial resolution, rich information, and resistance to water interference (Liedtke et al., 2021). However, Raman signal is weak and easily interfered by external factors such as fluorescence (Naqvi et al., 2022). Various Raman spectroscopy techniques are proposed to solve the weak signal and interference problem, such as Surface-enhanced Raman scattering (SERS), which overcomes the problems of weak Raman signal and fluorescence interference. Besides, Raman spectroscopy coupled with micro-imaging realizes the chemical compound visualization in the cell or metabolic levels, which assists in exploring the heavy metal stress process and migration rules in plants.

### Atomic spectroscopy

LIBS is an atomic emission spectroscopy technology that utilizes a focused pulsed laser beam to generate plasma from materials, then analyzes the elemental composition from the emission spectrum (Peng et al., 2016). The essential characteristics of LIBS are the ability to detect all elements, sample detection in different matrices, simultaneous multi-element detection ability, little or no sample preparation, real-time analysis, *in-situ* diagnosis, and remote detection (Jantzi et al., 2016). The moisture content in the material limits the detection ability of LIBS for the moisture content may reduce the emission intensity and affect the stability of the signal (Peng et al., 2017).

### Multitechnique spectroscopy

Chlorophyll fluorescence (CHI-FI) technology as a photosynthetic probe can reflect the distribution of heavy metal elements in plants, using chlorophyll as a judge. Zhang et al. (Zhang et al., 2022) and Das et al. (Das et al., 2015) combine spectroscopic techniques with chlorophyll fluorescence technology to detect and visualize the distribution of heavy metal elements such as Cd and Cu in plants. Therefore, multi-device/technology is considered an effective way to reveal the distribution of heavy metals and plant response at multiple scales.

Spectral data is repetitive, complex, and high dimensional, so machine learning methods are needed for effective data mining, such as dimensionality reduction, feature band extraction, modeling, etc. For HSI, RS, and CHI-FI imaging spectroscopy, traditional machine learning methods are challenging to meet the requirements, so it is necessary to use deep learning methods to process high-dimensional data. Detection of heavy metals in plants can be rapidly predicted using mathematical models with good performance.

## Basic steps of spectroscopic detection

**Figure 3:**
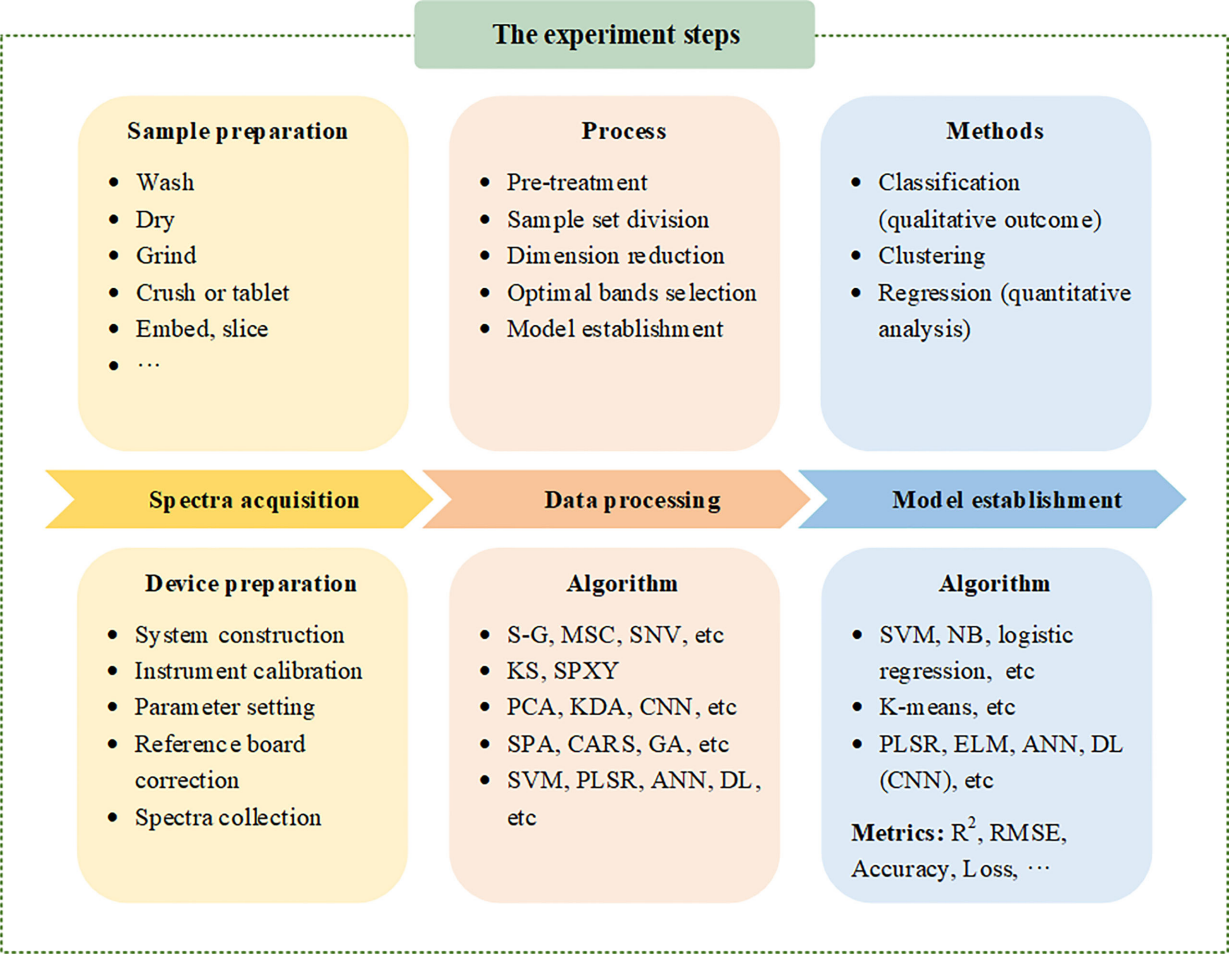
The major steps of the experiment are sample preparation, spectra acquisition and computational analysis.

The major steps of the experiment are sample preparation, spectra acquisition and computational analysis. The major steps of the experiment are shown in **Figure 3**.

### Spectrum acquisition

The first step of sample preparation is essential for spectrum acquisition. Usually, plant samples need to be cleaned, dried, and crushed (Li et al., 2016a), and many samples also need to be appropriately embedded and sliced. Drying, grinding, and pressing are usually preferred for dried samples using LIBS to acquire spectral (Peng et al., 2017; Peng et al., 2018). Appropriate embedding and slicing are necessary conditions for obtaining microstructure with spectral properties through Raman spectroscopy (Gierlinger et al., 2012; Liedtke et al., 2021).

An acquisition system is essential for spectrum acquisition, including a darkroom, light source, spectrometer, sample room and computer. For imaging spectrometers, a CCD camera is also required (Lowe et al., 2017). Spectral measurements were performed in a darkroom to control lighting conditions and reduce the effects of stray light (Wang et al., 2014). At the same time, attention should be paid to spectral resolution and determination range (Fu et al., 2020; Zhou et al., 2020b). When collecting spectra, the surrounding environment should be stable and corrected with a standard reference plate (Mishra, 2021).

### Data preprocessing

Computational processing is necessary to extract useful information from a large amount of data. Spectral data processing includes preprocessing, sample set division, dimensionality reduction, optimal band selection, and model establishment. For hyperspectral data, radiation calibration, atmospheric correction, geometric correction, and other preprocessing are also required before preprocessing (Li et al., 2012; Li et al., 2015). The original spectrum contains not only spectral information, but also noise signals that may interfere with spectral information due to various factors such as sample background and stray light (Mishra et al., 2017). Therefore, before data analysis, spectral preprocessing is used to remove noise and filter data, including Savizkg-Golag convolution smoothing (S-G), standard normal variable transform (SNV), multivariate scattering correction (MSC), short-time Fourier transform (STFT), Functional dependency (FD), fast Fourier transform (FFT) derivation, and wavelet transform (WT). Smoothing eliminates the noise in the spectrum; SNV reduces the influence of nonspecific scattering on particle surface (Wang et al., 2018a); MSC eliminates the scattering effect and enhances the spectral absorption information related to components; the modified baseline is derived and the wavelet transform is used for data denoising and smoothing (Wang et al., 2018a). Principal component analysis (PCA) and other methods can be used to reduce the dimension of spectral data (Pyo et al., 2020; Yu et al., 2020). At the same time, it is also necessary to select the best band, mainly including genetic algorithm (GA), uninformative variable elimination (UVE,and the successive projection algorithm (SPA) (Milanez et al., 2017).

### Machine learning

Machine learning has become increasingly popular in the critical field of spectroscopy (Han et al., 2022). When processing spectral data, spectral modeling methods can be further divided into the classification model (qualitative results) and the regression model (quantitative estimation) (Mishra et al., 2017). Classification is a supervised learning method used to identify the categories of different substances. Common methods include support vector machine (SVM), Least squares support vector machine (LS-SVM), naive Bayes (NB), decision tree, and logical regression. (Wang et al., 2019). Clustering is an unsupervised learning task, which can automatically classify samples, such as k-means, hierarchical clustering, and Gaussian mixture model.

Regression analysis predicts the value of variables by establishing linear or nonlinear models. A variety of machine learning methods are applied to regression analysis, such as partial least squares regression (PLSR) (Peng et al., 2016; Yu et al., 2020), extreme learning machine (ELM), and artificial neural networks (ANNs). (Melgani and Bruzzone, 2004; Li et al., 2018).

With the development of artificial intelligence, deep learning method has gradually become a research hotspot, which has shown the most advanced performance in image-based data processing (Li et al., 2021b). As a data-driven learning method, deep learning can automatically learn the low-dimensional and high-dimensional features contained in the data from the original data. It was initially suitable for two-dimensional image data, but its applicability has recently extended to one-dimensional spectral data. The Convolutional neural network (CNN) is one of the representative deep learning algorithms.

## Spectroscopy for detecting plants heavy metals

HMS influences the growth of plants and physiological parameters (such as chlorophyll content and enzymatic activity), leading to changes in reflection characteristics (Zhou et al., 2019a). Therefore, plant heavy metal levels and physiological parameters can be evaluated by analyzing spectral reflection information (Fu et al., 2020; Shen et al., 2020). Root, stem, and leaf play an essential role in plant growth, and reflect the growing situation. Plant roots absorb and enrich heavy metals and migrate them to stems and leaves. The research on detecting heavy metals in plant’s roots, stems, leaves, and fruits provides a theoretical basis for exploring the migration characteristics of heavy metals in plants. Recently, spectroscopic technology is extensively applied in analyzing plants’ leaves or roots to get the HMS level or growth information, and some research results have been obtained. The HSI, Vis-NIR, CHI-FI, LIBS, and RS are the most commonly used spectrum in acquiring plant information. This part mainly summarizes the research achievements of five spectroscopic technologies in detecting plants under HMS from different plant organs.

### HMS of roots

Roots are one of the vegetative organs of plants, which absorb water and inorganic salts in the soil and play the role of supporting, reproducing, and storing organic matter. HMS inhibits root growth, for example, by reducing the number of branches. Using spectral technologies to detect roots under HMS is more focused on traditional Chinese medicine plants or plants with strong soil remediation ability. **Table 1** shows examples of the application of spectroscopic techniques in root detection under heavy metal stress.

**Table 1:** Lists the application of spectral technology in the detection of roots under HMS.

Techniques	Plant (HMS)	Data analysis	Results	Ref.
CHI-FI, UV, AAS	Corn (Cd)	Chemometrics	R^2^ = 0.88	(Silva et al., 2012)
CHI-FI, Vis-NIR, AAS	Wheat, beans, vegetables (Cu)	GM,AM,LM	Spectral intensity∝Cu content of leaves	(Qu et al., 2012)
RS	Vetiver (Cu)	PLS	R^2^ = 0.78,	(Liu et al., 2015b)
LIBS, AAS	herbal medicine (Pb)	Internal standard method	R^2^ = 0.98	(Li et al., 2016a)
LIBS, AAS	Coptis chinensis (Cu)	Internal standard method	R^2^ = 0.99,	(Li et al., 2016b)
LIBS	Tobacco (Cd)	SPA; PLS,SVM	SVM(R^2^ = 0.98) >PLS (R^2^ = 0.94)	(Liu et al., 2018)
HIS, ICP-OES	Herb (Cd)	CARS; PLS, LS-SVM	CARS-LS-SVM(R^2^ = 0.9) >CARS-PLS (R^2^ = 0.87)	(Feng et al., 2019)
RS	Apple rootstock (Cu)	SG; SVM PLS-DA	SVM (Accuracy: 100%)>PLS-DA (Accuracy: 96%)	(Li et al., 2022)

**Figure 4:**
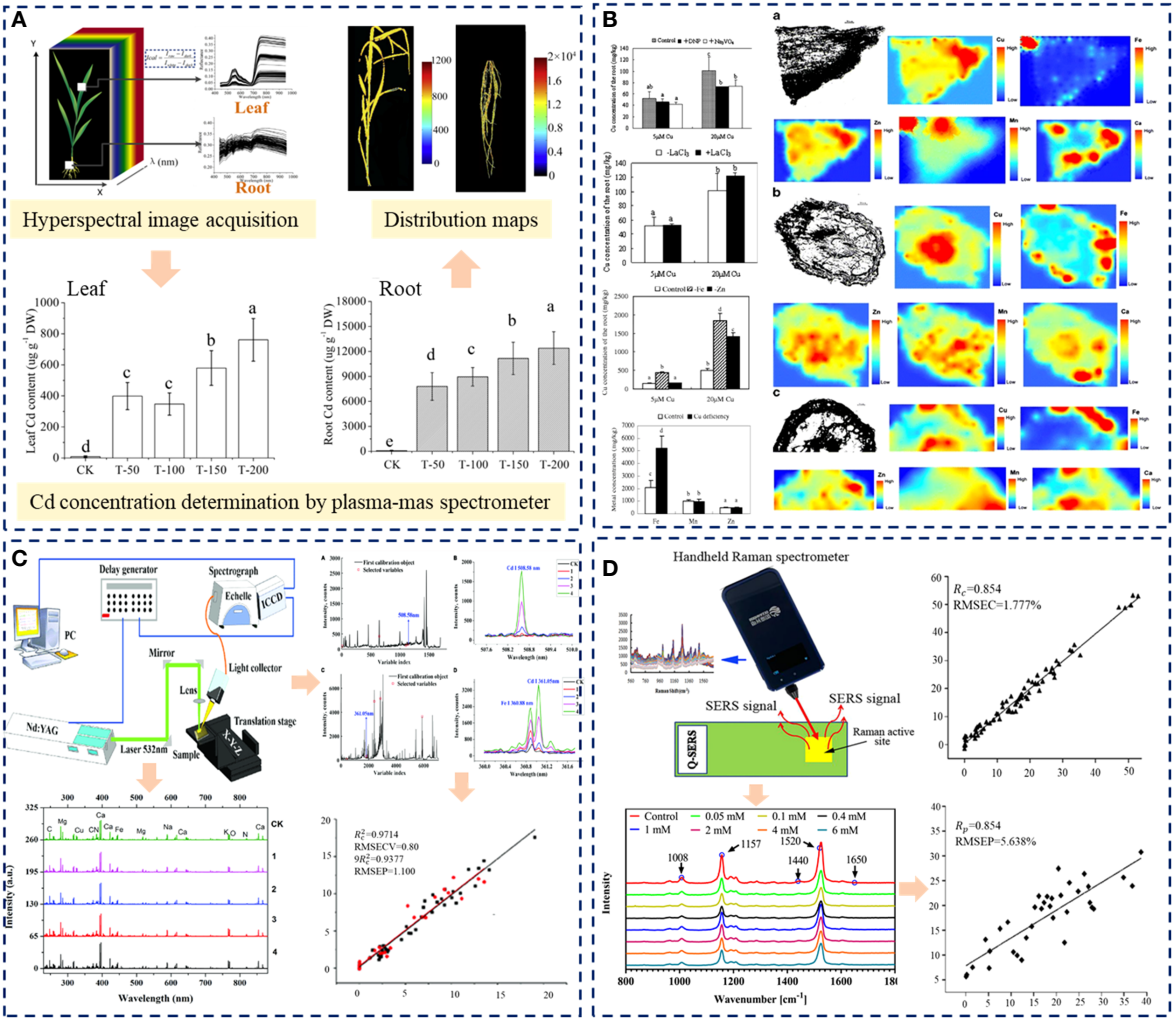
Application of spectral technology in detecting heavy metals in plant roots. **(A)** Rapid detection of cadmium and its distribution in Miscanthus sacchariflorus by visible and near-infrared hyperspectral imaging. Different letters (a, b, c, d, e.) in each histogram indicate a significant difference at P<0.01. **(B)** The effects of copper absorption and metal distribution in Commelina communis root growing area were analyzed by XRF. (a) Longitudinal section around the meristem and metal locations; (b) Cross section of elongation tissue and metal locations; (c) Cross- section of non-growing tissue and metal locations. The scale bar of sections represents 50μm. Different letters (a, b, c,d.) in each histogram indicate a significant difference at P<0.05. **(C)** Quantitative analysis of cadmium in tobacco roots was carried out by LIBS spectroscopy and stoichiometry. **(D)** Analysis of Raman spectroscopy in root detection of heavy metals. SERS, Surface-Enhanced Raman spectroscopy; XRF, X-Ray fluorescence; LIBS, Laser-induced breakdown spectroscopy.

The results showed that spectral technology combined with machine learning could detect heavy metal stress in herbaceous plants and roots. Qu et al. (Qu et al., 2012) acquired the apparent reflectance spectra of wheat, cabbage, and pea under Cu stress, and extracted their fluorescence spectra based on the model inversion method. The positive correlation between the height of the far-red fluorescence peak (FFR) and the Cu content in leaves was established. The extraction method of fluorescence spectrum based on the theoretical model proposed in this research provides a new method for this research field. Silva et al. (Silva et al., 2012) detected the accumulation mechanism of Cd in maize leaves and roots based on fluorescence spectroscopy. They monitored the chlorophyll and biomass changes, which proved the increase of Cd accumulation in roots could reduce the migration of metals to above-ground parts. The research illustrated that fluorescence spectroscopy has excellent sensitivity to the changes of plants under Cd stress, and can be used for early detection of heavy metal content in plants. Through the analysis of plants, it is further used for the identification of pollution environments and the monitoring of risk areas. Liu et al. (Liu et al., 2018) detected the Cd in tobacco root samples by LIBS, the best quantitative model was achieved by the IPLS-SVM model with R^2^ of 0.9820. This research provides a feasible, effective and economical approach for fast detecting Cd in tobacco roots. Li et al. (Li et al., 2016a; Li et al., 2016b) LIBS was used for rapid quantitative analysis of Cu in the roots of three Chinese herbal medicines and selected Pb 405.7 nm as the characteristic spectral line for analysis. The results showed that LIBS could be used for rapid detection and analysis of Cu in Chinese herbal medicines, and the internal standard method could improve the fitting accuracy. LIBS characteristic line extraction combined with machine learning can quickly predict heavy metal elements. Liu and Wang et al. (Liu et al., 2015b; Wang et al., 2017b) detected the contents of Cu and Pb in vetiver roots by Raman spectroscopy and analyzed the compounds represented by characteristic Raman bands. The results proved that the PLSR prediction model of heavy metal content after first-order differential pretreatment was optimal. In this research, indirect quantitative analysis of Cu content in resin after adsorption of heavy metals further confirmed the great potential and application value of vetiver in soil and water conservation and soil heavy metal remediation. **Figure 4** shows the application analysis of HSI, LIBS, CHI-FI, and RS spectral techniques in detecting heavy metals in plant roots. The spectrum obtained in **Figure 4** not only can determine heavy metals in plants, but also can visualize heavy metals and components of stressed organs by combining the spectrum with the image, which is helpful in studying the distribution and migration characteristics of heavy metals in plants.

It is challenging to degrade naturally in the soil polluted by heavy metals, which can only change and migrate the form. Using plants with strong heavy metal enrichment ability to transfer heavy metals in soil plays an essential role in the remediation of heavy metal contaminated soil. Phytoremediation is a set of methods to eliminate, destroy, metabolize, fix and stabilize heavy metal contaminates using plants. It is an effective way to control heavy metal contaminates in soil. Root exudates (such as citric acid and glycine) play a crucial role in phytoremediation. Generally, heavy metals can be absorbed by plant roots, harvested and concentrated on the ground, or filtered, fixed and, passivated by plant roots, to reduce their activity and pollution. Detecting the content of heavy metals in plant roots by spectroscopy is an assistant in explaining its accumulation mechanism, determine its effect, and selecting plants with strong heavy metal enrichment ability for soil remediation.

Currently, many researchers focus on the wrapping of heavy metals in roots to further reduce the damage of heavy metals in plants. Moreover, optical imaging spectroscopy provides a new way to evaluate the heavy metals’ adsorption capacity of plants and resistant breeding.

### HMS of stems

The stem is one of the vegetative organs of plants, transporting nutrients and water to support leaves, flowers, and fruits. The stems of some plants also have the functions of photosynthesis, storage of nutrients, and reproduction. Many scholars have found the accumulation and distribution of heavy metals in plant stems. However, spectroscopic analysis of changes in plant stems components under HMS is also required. Zhang et al. (Zhang et al., 2019a) used fluorescence spectroscopy to detect the Cd content in Chinese herbal medicine rapidly and established the calibration curve of Cd content and fluorescence intensity. The detection limit of Cd was 0.083 mg/kg, and the quantitative limit was 0.207 mg/kg. The accuracy was similar to that of chemical analysis, and the detection time was short, which could be used to monitor heavy metals in Chinese herbal medicine manufacturers. By near-infrared spectroscopy, Xu et al. (Xu et al., 2011) quickly predicted the contents of Cd, Cu, and Zn in sweet potato leaves and stems. Partial least squares regression (PLS) was used to model and analyze the contents of water, protein, Cd, Cu, and Zn in sweet, potato, leaves and stems. The results show that near-infrared spectroscopy can be used to quickly predict the contents of Cd, Cu and Zn in sweet, potato, leaves, and stems. Plant inorganic ions can be combined with organic groups with near-infrared absorption in a specific form, so that Vis-NIR can detect their content. However, the Vis-NIR has high requirements for the representativeness and classification of the calibration model sample group and the uniformity of the sample size, so it is necessary to explore suitable model optimization methods.

The stem of the plant has a transduction effect. It can transport the water and inorganic salts absorbed by the root system from the soil to the leaves, flowers, fruits, and other ground parts. Stems are accompanied by heavy metal migration when transporting water, inorganic salts, and other substances. Therefore, studying the heavy metal contaminantes of plant stems is necessary. However, spectral technology is more used to detect the content or phenotype of plant leaves or roots under HMS, but the corresponding mechanism of stems is less researched. More research can be done in this field in the future, which will help to understand the migration characteristics of heavy metals in plants.

### HMS of leaves

As an essential organ of plants, leaves synthesize organic matter through photosynthesis, supply essential nutrients for plants growth, and promote for roots to absorb water and mineral nutrients from the outside through transpiration. Leaves are crucial indicators that reflect the biochemical composition and health status of vegetation. Getting physiological information about leaves is important to understand plant characteristics (Fu et al., 2020). In particular, detecting leaf physiological information under heavy metal stress by spectral technology is helpful in researching the corresponding mechanism of plants under heavy metal stress. **Table 2** lists the application of spectral technology in detecting leaves under heavy metal stress. The results show that spectral technology combined with machine learning and chemical methods has excellent advantages in the detection of heavy metals, and it is a rapid method for the detection of heavy metals in plants.

**Table 2:** Application of spectral technologies in detecting the quality of the leaves under HMS.

Techniques	Plant (HMS)	Data analysis	Results	Ref.
Vis-NIR	*Ludwigia Prostrata*	PLSR	R=0.950	(Liu et al., 2012)
HSI, LIBS	Tobacco (Cu)	Calibration curve	R^2^ = 0.98, LOD:7.72 mg/kg	(Lu et al., 2017)
LIBS	Rice (Cr)	PLSR	R^2^ = 0.97, LOD:4.75mg/kg	(Peng et al., 2017)
LIBS, ASV	Vegetables (Cd)	SNV, FD, SD, CT; PLSR	The best method: CT, R=0.99	(Yang et al., 2017)
Vis-NIR	Corn (Cu)	Regression	R^2^ = 0.73,RMSE=0.013	(Li et al., 2019)
Vis-NIR	Tomato (Cd)	WT; LSSVR	R^2^ = 0.94, RMSE=0.0099	(Jun et al., 2019)
Vis-NIR	Lettuce (Cd)	WT, SD, PCA, VISSA,GOA; SVM	The best method: VISSA, GOA; Accuracy:98.57%	(Zhou et al., 2019b)
HSI	Corn (Cu)	OIF-PLS	R=0.85	(Gao et al., 2020)
HSI	Rice (Cd)	GA, CARS; PLS, ELM, LS-SVM	The best method: CARS; ELM, R=0.94	(Shen et al., 2020)
HSI	Lettuce (Cd, Pb)	WT, SCAE;DL, SVR	Cd: R^2^ = 0.93, RMSE=0.050 Pb: R^2^ = 0.94, RMSE=0.041	(Zhou et al., 2020b)
HSI	Tobacco (Hg)	PCA, CARS; PLS-DA, LS-SVM	The best method: CARS; LS-SVM, Accuracy:100%	(Yu et al., 2021)
Vis-NIR	Corn(Cu)	STFT, PLSR	R^2^ = 0.99	(Meng et al., 2021)

**Figure 5:**
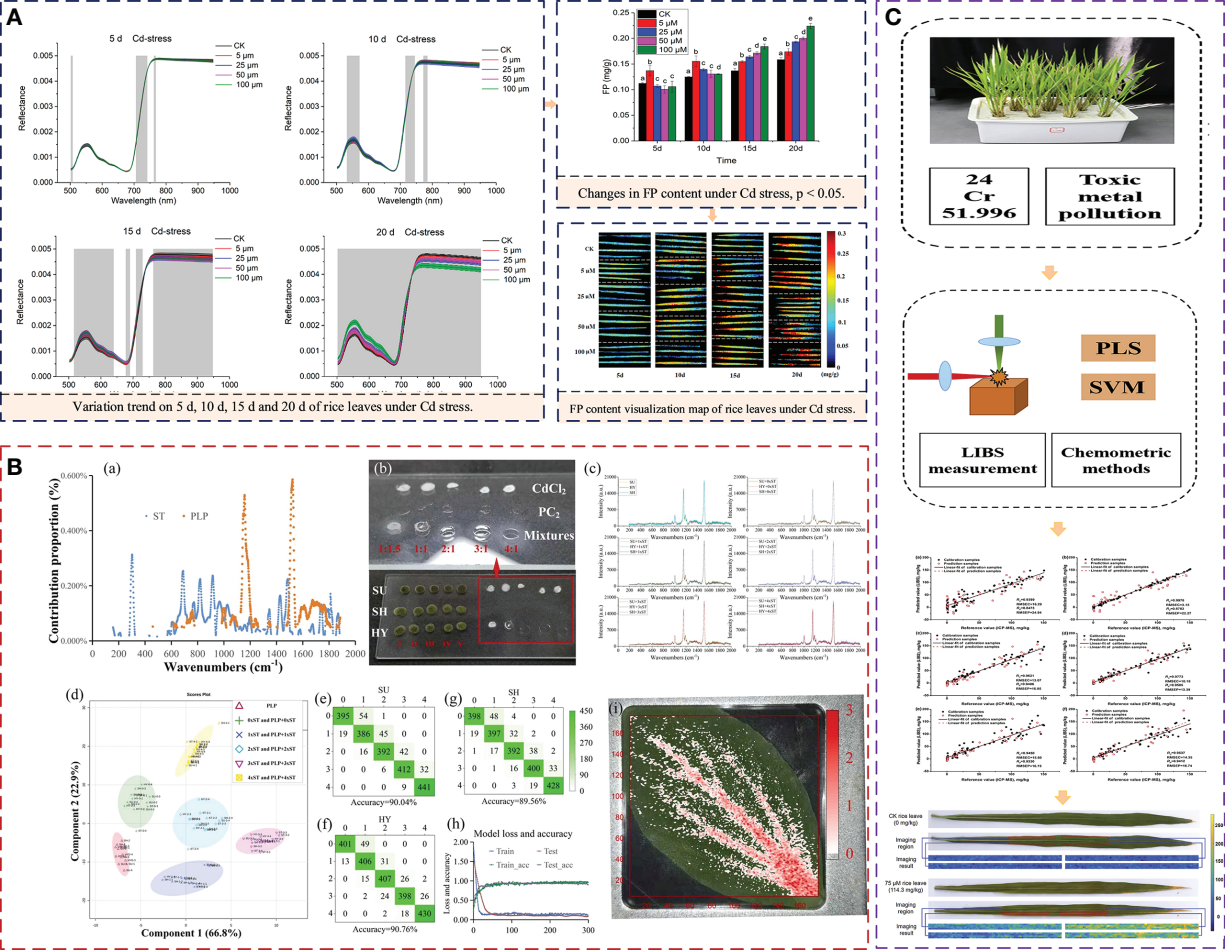
Spectrum processing process and flow chart. **(A)** Flowchart of image processing and data analyses for predicting the FP in rice leaves. Different letters (a, b, c, d, e.) in each histogram indicate a significant difference at P<0.01. **(B)** Raman spectrum analysis results of leaf tissue of Pakchoi under heavy metal cadmium stress. **(C)** Optimization method for rapid visualization of chromium distribution in rice leaves by LIBS spectroscopy. FP, free proline; ST, standard; PLP, pure leaf powders; PLS, partial least squares; SVM, support vector machine; LIBS, Laser-induced breakdown spectroscopy.

Shen et al. (Shen et al., 2020) used HSI to conduct high-throughput screening of free proline (FP) in rice leaves under Cd stress and established PLS, LS-SVM, and ELM models based on effective wavelength, realizing the FP content visualization. The results showed that ELM with 27 sensitive wavelengths had the best performance for predicting FP detection. Hyperspectral imaging and machine learning can rapidly and non-destructively estimate the physiological parameters of leaves, providing a technical means for real-time and high-throughput screening of plant phenotypic physiological parameters under heavy metal stress. Fu et al. (Fu et al., 2020) recognized Cu and Pb in Maize Leaves Based on HSI and analyzed the recognition characteristics of Cu and Pb in the frequency domain. The results show that the red edge and red shoulder region of the spectrum can be used as the characteristic spectrum to distinguish Cu and Pb. This research demonstrates the potential of frequency domain in identifying small spectral differences. The concentration of heavy metals in plants can be obtained by analyzing the spectra of plants, and further indirectly estimating the concentration of heavy metals in water and soil. Peng et al. (Peng et al., 2018) established the Cr prediction model of rice leaves based on global spectra after the optimization of two important parameters (delay time and energy ratio) in dual-pulse laser-induced breakdown spectroscopy (DP-LIBS), with a correlation coefficient of prediction of 0.959 and the distribution of chromium in rice leaves were visualized with the best prediction model. Feng et al. (Feng et al., 2019) also realized the visualization of Cd concentrations in *Miscanthus sacchariflorus* based on HSI. Zhao et al. (Zhao et al., 2022) did the structure analysis and non-invasive detection of cadmium-phytochelatin2 complexes in the plant by deep learning Raman spectrum. Phytochelatin2 (PC_2_) chelates Cd^2+^ in a 2:1 ratio to form Cd (PC_2_)_2_; Cd-S bonds of the Cd (PC_2_)_2_ have signature Raman vibrations at 305 and 610 cm^−1^ are the most distinctive spectral signatures for Cd-PCs complexes. This research provides a general protocol using Raman information for structure analysis and non-invasive detection of heavy metal-PCs complexes in plants. It provides a novel idea for simplifying the identification of high phytoremediation cultivars and assessing heavy metal-related food safeties. **Figure 5** displays the typical common spectra application in detecting plant leaves under heavy metal stress. The figure shows the process of heavy metal detection and analysis, spectrum acquisition, data preprocessing, modeling, and visual expression.

However, it is difficult to extract features manually by using machine learning methods to process spectral data, and the issues of such considerable data in spectral data increase the spectral processing load, leading to the complexity of spectral feature extraction and mathematical modeling. Deep learning shows obvious advantages in processing massive data. Applying it to processing spectral data is conducive to extracting data features and improving the detection and recognition rate. Zhou et al. (Zhou et al., 2020b) developed a deep learning method based on wavelet transform (WT) and stack convolution auto encoder (SCAE) to detect the depth characteristics of lettuce leaves under heavy metal stress. The performance of Support vector machine regression (SVR) model based on depth features obtained by WT-SCAE is reasonable, and the predictive decision coefficient (R_p_^2^) is 0.9319. This research confirms the great potential of combining hyperspectral techniques with deep learning algorithms for detecting composite heavy metals.

As an essential part of plants, leaves can reflect the growth and physiological state of plants. Using spectral technology to detect the leaves of plants under heavy metal stress can obtain the influence of heavy metals on the phenotypic information of leaves and the concentration information of heavy metals. The research on the leaf information of plants under HMS is mostly the detection of heavy metals in food plants or leaf vegetables, which is of great significance to food safety. However, most of the current researches are based on laboratory conditions to carry out single heavy metal stress on leaves and use spectral technology to quantitatively detect heavy metal content in leaves or analyze physiological information of plants. The spectral analysis also mainly focuses on constructing vegetation indices to obtain leaf physiological information. For detecting HMS of large-scale farmland plants, portable detection equipment and online monitoring equipment based on spectral technology is still the focus of research and development, and the critical point of digital agriculture implementation. Researchers are more likely to combine hyperspectral imaging technology with remote sensing technology and use hyperspectral remote sensing technology to detect heavy metal content in large-area planted plants, which also provides the possibility for real-time and efficient monitoring of heavy metal contaminations. In the future, it may play an essential role in providing key information in space and time for precision agriculture.

### HMS of fruits

Fruit is closely related to human life. In human food, most of them are the fruits of gramineous plants, such as wheat, rice, and corn. People often eat fruits, including apples, peaches, oranges, and grapes. They are rich in glucose, fructose, sucrose, various and inorganic salts, vitamins and other nutrients. These fruits are not only delicious, but also processed into dried fruit, jam, preserves, wine, fruit juice, and vinegar. In addition, some fruits also have certain medicinal value, such as jujube, fennel, papaya, citrus, hawthorn, apricot and longan, which can be made into Chinese herbal medicine. Therefore, nondestructive fruit quality testing is conducive to ensure its commercial value and food safety. Currently, many studies have been conducted to detect the quality of fruits Based on spectral technology, but there are few studies on detecting heavy metals in fruits.

Lin et al. (Lin et al., 2014) analyzed the cadmium content in Gannan navel orange based on LIBS technology and compared it with the analysis results of AAS technology after wet acid decomposition. The correlation coefficients of LIBS and AAS analysis results were 0.9096 and 0.991, respectively, with little difference between them. Food is indispensable for human survival, it is essential to understand its toxic trace level to determine its potential impact on human health. As a non-destructive testing technology, spectral technology has significant advantages in the rapid non-destructive testing of fruits. LIBS technology is expected to play an essential role in detecting heavy metals in fruits because it does not require complex sample preparation.

### Others

The canopy is a dense top layer of trees. As the first part of the plant to contact with the external gas environment and light, plant canopy is related to the use of light energy, such as light transmission, reflection, photosynthesis, and transpiration rate. It can be used to evaluate the growth of plants. The detection and evaluation of heavy metal content in canopy by spectral technology play an important role in food safety and environmental monitoring. Scholars have used spectral technology to detect and analyze plant canopy under heavy metal stress and explored the spectral effect of heavy metal stress on plants. Shi et al. (Shi et al., 2016) collected the field canopy reflectance spectra in the jointing-booting growth stage of rice, and selected the well-performing vegetation indices using successive projections algorithm (SPA), then adopted the SPA selected vegetation indices to calibrate a multiple linear regression model for estimating soil arsenic content. Results showed that a three-band vegetation index performed best in estimating content. The vegetation index can be effectively used to estimate the content of heavy metals in plants, the combination of characteristic wavelengths in spectral information is helpful in improving the estimation accuracy. Liu et al. (Liu et al., 2015a) realized the dynamic simulation of rice growth parameters in cadmium contaminated soil to monitor the stress-induced changes of growth parameters on the time scale. Results showed that the growth parameters simulated by the modified WOFOST model reflected the variations of rice growth status on a time scale. This research provides a reference for dynamically monitoring heavy metal contamination in farmland environments. Kancheva et al. (Kawamura et al., 2021) obtained the canopy reflectance data of alfalfa, spring barley, and pea under Cd and Ni stress, studied the sensitivity of plant spectral response (various vegetation indexes and red edge positions) to pollution level and crop performance (growth variables), and obtained the relationship between heavy metal stress and plant phenotype through regression analysis. In addition, the transmittance and chlorophyll fluorescence excited by blue light (470nm) were measured on isolated leaves, statistically correlated with plant pigment content and cadmium concentration. The results showed the stress growth conditions were caused by significant plant spectral response changes. Various spectral characteristics were highly correlated with stress factors. This also provides a theoretical basis for using spectral technology to describe plant physiological development and ecological tranquility. Zhang et al. (Zhang et al., 2019b) used fluorescence spectroscopy to determine the content of heavy metals As, Pb, and Hg in edible roses. The detection limits were 0.0053, 0.0227, and 0.0079 μg/L, respectively. It was found that the content of heavy metals in edible roses exceeded the standard. The results showed that the detection of heavy metal elements as, Pb, and Hg in edible roses by fluorescence spectroscopy could meet the detection requirements, and it was expected to be used to determine the heavy metal content of edible flowers such as canary flowers, bitter thorn flower, and pear flower. There are few studies on detecting heavy metal content in flowers by spectral technology, and the research methods need to be further studied. Wang et al. (Wang et al., 2017a) collected the spectral data of wheat canopy at different growth stages under the stress of heavy metals Cu and Zn and explored the effects of Cu and Zn stress on the spectra of wheat. The results showed that heavy metals had different effects on the spectra of wheat at different growth stages. The red trough position (650nm) in the canopy spectral characteristics would move to the short-wave direction, that is, the blue shift phenomenon occurred, and the green peak position (550nm) would move to the long-wave direction, that is, the redshift phenomenon occurred. The red edge blue shift phenomenon of spectral characteristics is also related to the change of chlorophyll content of plants under heavy metal stress.

It is feasible to detect plant canopy under heavy metal stress by spectral technology, which can be used to study the response mechanism of plants to heavy metal stress, especially through the construction of vegetation index. At the same time, spectral technology combined with remote sensing technology can be used to realize large-area Farmland Monitoring and biological parameter inversion of plant canopy under heavy metal stress. With the continuous development of sensor technology and computer technology, it is more potential to realize the detection of trace heavy metals in plants and the small changes of plant physiological indexes based on spectral analysis.

## Conclusion and prospect

With the development of optical instruments and machine learning, applying optical imaging spectroscopy to the real-time detection of heavy metals in plants has become a possibility. At the same time, optical imaging spectroscopy can reveal plant phenotypic information at multiple scales, making the distribution of heavy metals visualized in multiple dimensions, and providing a new method to explore the migration pattern of heavy metals in plants. Moreover, the development of artificial neural networks has solved the problem of complex and tedious data mining in spectroscopy, further improved the sensitivity of spectra and reduced the detection limit.

Although optical imaging spectroscopy coupled with machine learning has proven to be a reliable means for plant heavy metal detection due to its rapid, high-throughput, simple operation, in-suit and real-time, it still faces some challenges: (a) Limited by the optical properties of plants, the sensitivity of optical imaging spectroscopy detection is lower than that of traditional chemically invasive analytical methods, thus seeking multiple devices/techniques coupled with spectroscopy to improve precision of optical instruments is the key to improve the sensitivity of spectroscopy. (b) Despite the simplicity of optical imaging spectroscopy detection steps, expensive and large equipment instruments are still the major constraints to the application of spectroscopy to plant heavy metal detection, so the development of portable and low-cost optical devices is the basis for promoting the industrialization of spectroscopy. (c) Improving the artificial neural network model and developing more algorithms guarantee full mining of spectral data, which is conducive to the further promotion of spectroscopy in phytoremediation and breeding.

## Author contributions

JL: writing – original draft and writing – review and editing. JR, and RC : investigation, writing – review and editing. KY: resources, and writing - review and editing. YZ: investigation, research, resources, and writing - review and editing. All authors read and approved the final manuscript.

## Funding

This work was supported by the National Natural Science Foundation of China (Program Nos: 31901403), Natural Science Foundation of Shaanxi Province of China (Program No. 2022JM-100), and the Key Laboratory of Agricultural Internet of Things, Ministry of Agriculture and Rural Affairs, P. R. China.

## Conflict of interest

The authors declare that the research was conducted in the absence of any commercial or financial relationships that could be construed as a potential conflict of interest.

## Publisher’s note

All claims expressed in this article are solely those of the authors and do not necessarily represent those of their affiliated organizations, or those of the publisher, the editors and the reviewers. Any product that may be evaluated in this article, or claim that may be made by its manufacturer, is not guaranteed or endorsed by the publisher.
